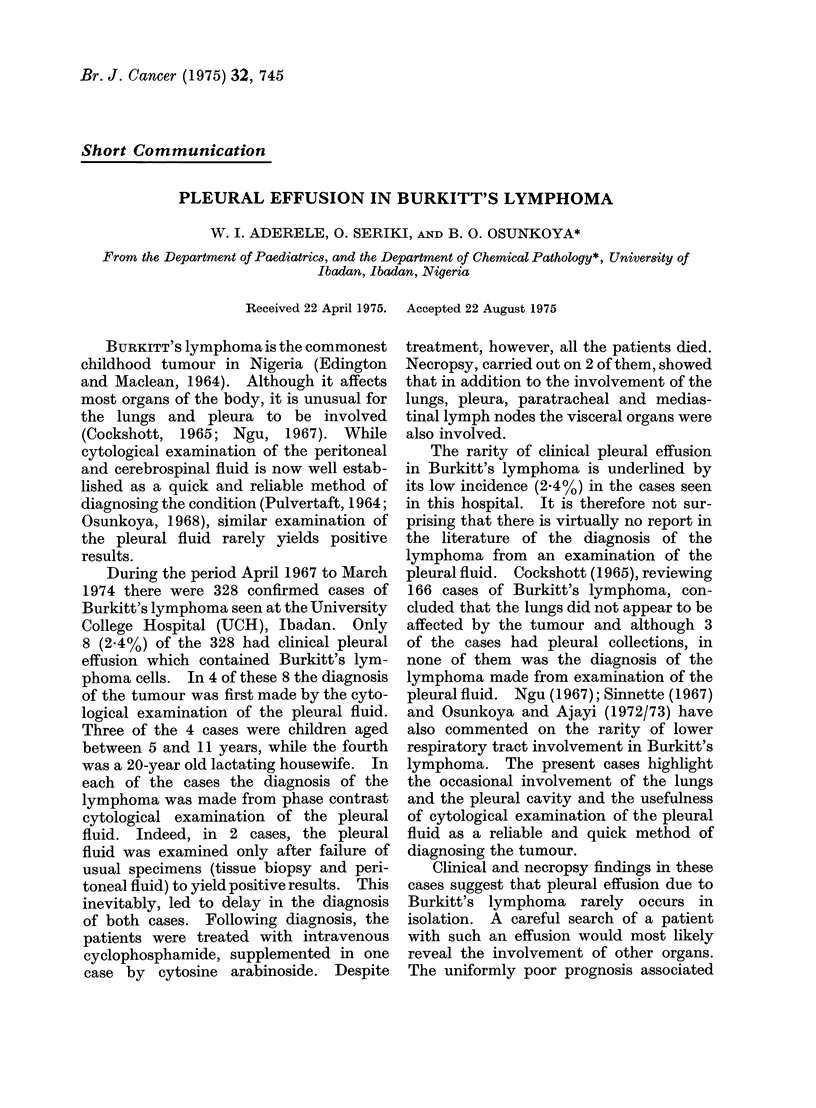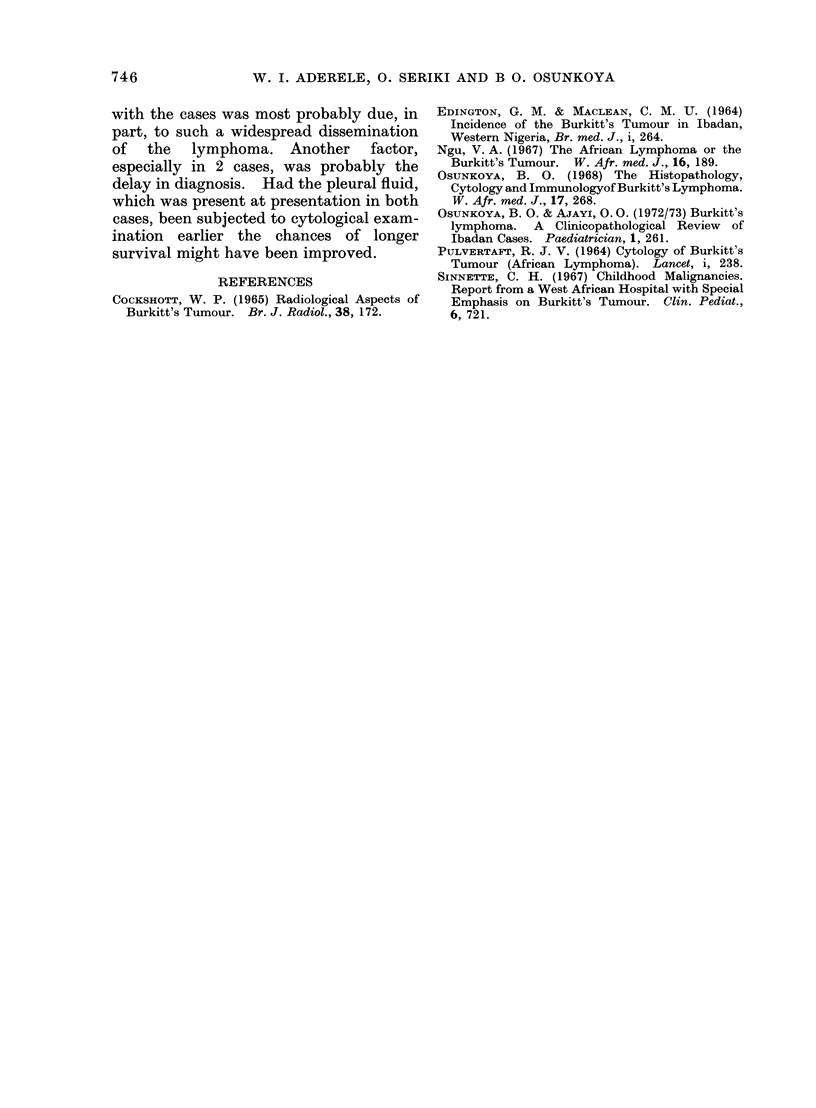# Pleural effusion in Burkitt's lymphoma.

**DOI:** 10.1038/bjc.1975.287

**Published:** 1975-12

**Authors:** W. I. Aderele, O. Seriki, B. O. Osunkoya


Br. J. Cancer (1975) 32, 745

***Short Communication***

### PLEURAL EFFUSION IN BURKITT'S LYMPHOMA

W. I. ADERELE, O. SERIKI, AND B. O. OSUNKOYA*

*From the Department of Paediatrics, and the Department of Chemical Pathology*, University of Ibadan, Ibadan, Nigeria*

Received 22 April 1975. Accepted 22 August 1975

BURKITT's lymphoma is the commonest childhood tumour in Nigeria (Edington and Maclean, 1964). Although it affects most organs of the body, it is unusual for the lungs and pleura to be involved (Cockshott, 1965; Ngu, 1967). While cytological examination of the peritoneal and cerebrospinal fluid is now well established as a quick and reliable method of diagnosing the condition (Pulvertaft, 1964; Osunkoya, 1968), similar examination of the pleural fluid rarely yields positive results.

During the period April 1967 to March 1974 there were 328 confirmed cases of Burkitt's lymphoma seen at the University College Hospital (UCH), Ibadan. Only 8 (2·4%) of the 328 had clinical pleural effusion which contained Burkitt's lymphoma cells. In 4 of these 8 the diagnosis of the tumour was first made by the cytological examination of the pleural fluid. Three of the 4 cases were children aged between 5 and 11 years, while the fourth was a 20-year old lactating housewife. In each of the cases the diagnosis of the lymphoma was made from phase contrast cytological examination of the pleural fluid. Indeed, in 2 cases, the pleural fluid was examined only after failure of usual specimens (tissue biopsy and peritoneal fluid) to yield positive results. This inevitably, led to delay in the diagnosis of both cases. Following diagnosis, the patients were treated with intravenous cyclophosphamide, supplemented in one case by cytosine arabinoside. Despite treatment, however, all the patients died. Necropsy, carried out on 2 of them, showed that in addition to the involvement of the lungs, pleura, paratracheal and mediastinal lymph nodes the visceral organs were also involved.

The rarity of clinical pleural effusion in Burkitt's lymphoma is underlined by its low incidence (2·4%) in the cases seen in this hospital. It is therefore not surprising that there is virtually no report in the literature of the diagnosis of the lymphoma from an examination of the pleural fluid. Cockshott (1965), reviewing 166 cases of Burkitt's lymphoma, concluded that the lungs did not appear to be affected by the tumour and although 3 of the cases had pleural collections, in none of them was the diagnosis of the lymphoma made from examination of the pleural fluid. Ngu (1967); Sinnette (1967) and Osunkoya and Ajayi (1972/73) have also commented on the rarity of lower respiratory tract involvement in Burkitt's lymphoma. The present cases highlight the occasional involvement of the lungs and the pleural cavity and the usefulness of cytological examination of the pleural fluid as a reliable and quick method of diagnosing the tumour.

Clinical and necropsy findings in these cases suggest that pleural effusion due to Burkitt's lymphoma rarely occurs in isolation. A careful search of a patient with such an effusion would most likely reveal the involvement of other organs. The uniformly poor prognosis associated with the cases was most probably due, in part, to such a widespread dissemination of the lymphoma. Another factor, especially in 2 cases, was probably the delay in diagnosis. Had the pleural fluid, which was present at presentation in both cases, been subjected to cytological examination earlier the chances of longer survival might have been improved.

### REFERENCES

Cockshott, W. P. (1965) Radiological Aspects of Burkitt's Tumour. *Br. J. Radiol.*, **38**, 172.

Edington, G. M. & Maclean, C. M. U. (1964) Incidence of the Burkitt's Tumour in Ibadan, Western Nigeria, *Br. med. J.*, i, 264.

Ngu, V. A. (1967) The African Lymphoma or the Burkitt's Tumour. *W. Afr. med. J.*, **16**, 189.

Osunkoya, B. O. (1968) The Histopathology, Cytology and Immunologyof Burkitt's Lymphoma. *W. Afr. med. J.*, **17**, 268.

Osunkoya, B. O. & Ajayi, O. O. (1972/73) Burkitt's lymphoma. A Clinicopathological Review of Ibadan Cases. *Paediatrician*, **1**, 261.

Pulvertaft, R. J. V. (1964) Cytology of Burkitt's Tumour (African Lymphoma). *Lancet*, i, 238.

Sinnette, C. H. (1967) Childhood Malignancies. Report from a West African Hospital with Special Emphasis on Burkitt's Tumour. *Clin. Pediat.*, **6**, 721.